# Multi-substrate chromosome preparations for high throughput comparative FISH

**DOI:** 10.1186/1472-6750-6-20

**Published:** 2006-03-20

**Authors:** Robert Hasterok, Joanna Dulawa, Glyn Jenkins, Mike Leggett, Tim Langdon

**Affiliations:** 1 Department of Plant Anatomy and Cytology, Faculty of Biology and Environmental Protection, University of Silesia, Jagiellonska 28, 40-032 Katowice, Poland; 2 Institute of Biological Sciences, Edward Llwyd Building, University of Wales Aberystwyth, Penglais, Aberystwyth, Ceredigion SY23 3DA, UK; 3 Institute of Grassland and Environmental Research, Plas Gogerddan, Aberystwyth, SY23 3EB, UK

## Abstract

**Background:**

A modification of a standard method of fluorescence *in situ *hybridisation (FISH) is described, by which a combination of several substrates and probes on single microscope slides enables more accurate comparisons of the distribution and abundance of chromosomal sequences and improves the relatively low throughput of standard FISH methods.

**Results:**

The utility and application of multi-colour, multi-substrate FISH is illustrated by the simultaneous physical mapping of retrotransposon sequences to three species of *Avena*, and single locus BAC (bacterial artificial chromosome) clones and rDNA probes to three species of *Brachypodium*, demonstrating how this would enable better understanding of complex phylogenetic relationships among some of the species belonging to these two genera.

**Conclusion:**

The results show that use of multi-substrate chromosome preparations significantly increases the utility of FISH in comparative analyses of the distribution and abundance of chromosomal sequences in closely related plant species.

## Background

Fluorescence *in situ *hybridisation (FISH) is used extensively and routinely in plant genome analysis (an excellent kaleidoscope of representative papers describing relevant techniques has recently been published [1]). It is particularly useful for applications such as comparing genome organisation in series of species or accessions (e.g. [2,3]) or identifying introgressions (e.g. [4,5]) where relatively large numbers of samples may be desirable. A major limitation in such studies is the low capacity of FISH methods, especially when compared to the high throughput of complementary molecular methods, such as Southern hybridisation and PCR. This limitation is further exacerbated by the need to make a number of duplicate experiments to control for variation between slides when comparing differential FISH signals. An approach that has been widely though infrequently used for comparative FISH is to combine substrates on a single slide [4,6-8]. Although far more exacting controls are required for accurate quantification [9], this approach does enhance reproducibility and allows a greater number of samples to be processed per slide. Here we present an optimised, combined substrate method suitable for both monocot and dicot plant species.

## Results and discussion

**Figure 1:**
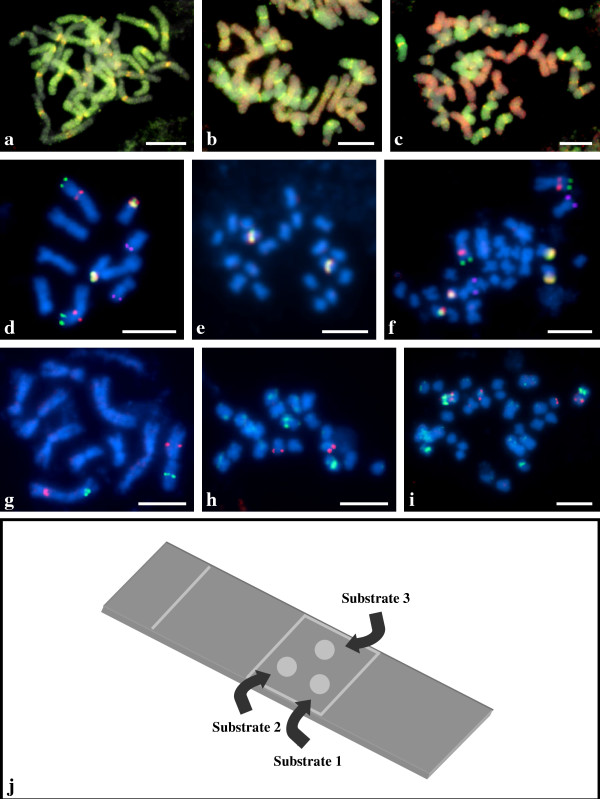
**(a-i) **Multicolour FISH applied to triple substrate chromosome preparations. **(a-c) **Retrotransposon-based probes KK2.5 (yellow fluorescence), KK3.4 (green fluorescence) and KK4.24 (red fluorescence) hybridising to somatic metaphase chromosomes of **(a) ***Avena agadiriana *(2n = 4x = 28; genomes AABB), **(b) ***A. murphyi *(2n = 4x = 28; genomes AACC) and **(c) ***A. sterilis *(2n = 6x = 42; genomes AACCDD). **(d-f) **BAC clone-based probes ABR1-41-A8 (green fluorescence) and ABR1-47-F4 (purple pseudocolour), ABR1-59-F9 (red fluorescence) and 25S rDNA-based probe (yellow fluorescence) hybridised to somatic metaphase chromosomes of **(d) ***Brachypodium distachyon *(2n = 2x = 10), **(e) **unidentified, presumably diploid species of *Brachypodium *ABR114 (2n = 2x = 20) and **(f) **an interspecific hybrid ABR113 (2n = 4x = 30) containing the two genomes shown in **(d, e)**. **(g-i) **BAC clones ABR1-41-E10 (green fluorescence) and ABR5-1-H3 (red fluorescence) hybridising to somatic metaphase chromosomes of **(g) ***B. distachyon*, **(h) **ABR114 (2n = 2x = 20) and **(i) **ABR113 (2n = 4x = 30). **(j) **Diagram of a triple substrate chromosome preparation. Scale bar: **(a-c) **10 μm, **(d-i) **5 μm.

Two factors were found to be of critical importance for the success of multi-substrate hybridisation experiments, namely the conditions used for enzymatic digest of each species and the conditions used for placing the substrates onto slides. Provided that these conditions are optimised, we find that separate distribution of the various substrates can be reliably achieved, allowing comparison even of samples with similar or identical karyotypes (such as *A. agadiriana *and *A. murphyi *in Fig. 1a, b). The optimal time of enzymatic digestion is that which allows intact meristems to be easily extruded from the root tip. Shorter times prevent complete removal of surrounding tissues such as root epiderm and calyptra, which compromise the quality of the chromosome spread, while longer times may result in disintegration of meristems and subsequent dispersal of chromosomes across the slide when cover slips are applied. Optimal digest times must be established for each species separately.

It is also necessary to transfer each of the substrates in a small volume, no more than 4 μl, and to dispense them so that each substrate is approximately the same distance from the nearest edge of the cover slip (i.e. for three samples, in the form of triangle as shown in Fig. 1j, for four samples, in the form of a rhombus). These arrangements minimise displacement and possible loss of material when applying the cover slip. We found 24 mm × 24 mm cover slips most suitable for making triple-substrate preparations. It is difficult to achieve well distributed but non-overlapping spreads of samples if smaller cover slips are used, while use of longer, rectangular cover slips impedes even spreading of acetic acid, frequently leading to incorporation of air bubbles.

### Triple substrate chromosome preparation of *Avena*

Cultivated oat, *Avena sativa*, is a hexaploid made up of two genomes (A and D) which are closely related, plus a third (C) that may be easily distinguished from the first two by GISH (genomic *in situ *hybridisation) using total nuclear DNA from wild *Avena *species. The A and D genomes have only recently been distinguished by FISH [10] and it is of interest to clarify their relationship to genomes of wild species. Root tip meristems from three different species of *Avena *(the allotetraploids *A. agadiriana*, *A. murphyi *and the allohexaploid *A. sterilis*) were used for triple substrate chromosome preparations and subjected to multicolour FISH with a mixture of three retrotransposon sequences. Probe KK2.5 (yellow/orange fluorescence) is restricted to centromeric regions of chromosomes belonging to both of the two genomes in *A. agadiriana *(Fig. 1a), one of the two genomes in *A. murphyi *(Fig. 1b) and two of the three genomes in *A. sterilis *(Fig. 1c). Probe KK3.4 (green fluorescence) labels intensely only one of the two genomes in *A. agadiriana *(Fig. 1a), to a slightly lesser extent, one of the two genomes in *A. murphyi *(Fig. 1b), and one of the three genomes in *A. sterilis *(Fig. 1c). Probe KK4.24 (red fluorescence) is totally absent from the chromosomes of *A. agadiriana *(Fig. 1a) but paints intensively the chromosomes of one genome in both *A. murphyi *(Fig. 1b) and in *A. sterilis *(Fig. 1c). Thus, *A. agadiriana *is seen to be an AB tetraploid and lacks the C-genome specific KK4.24 target, and *A. sterilis *is an ACD hexaploid, with the A and D genomes differentiated by the KK3.4 probe. The use of multi-substrate FISH allows an accurate comparison to be made of the extent of hybridisation of the KK3.4 probe, so that it can easily be seen that the non-C genome in *A. murphyi *shows levels of the KK3.4 target intermediate between the *A. sterilis *A and D genomes, indicating that it is not a recent progenitor of *A. sterilis *or, by extension, cultivated hexaploid oat.

### Triple substrate chromosome preparation of *Brachypodium*

Wild tetraploid and hexaploid accessions of *B. distachyon *were found which formed a series based upon 2n = 2x = 10, and were proposed to be autopolyploids [11]. However, our comparative GISH analyses [12] indicated that accession ABR113 is in fact an allotetraploid containing two genomes similar or identical to those in two diploid species previously described as ABR1 and ABR114 accessions of *B. distachyon*. Multiple substrate FISH allowed us to investigate this further. Figure 1d-i shows distribution of selected clones of a *B. distachyon *BAC library landed in chromosomes of *B. distachyon *and two closely related species. The 41-A8 clone (green fluorescence) hybridises to the distal part of one arm of the largest and metacentric pair of chromosomes in the complement, while 59-F9 (red fluorescence) gives well defined signals in the proximal and pericentromeric part of the same arm. The 47-F4 clone (purple pseudocolour) maps to the distal part of the short arm on small subterminal chromosomes, while the 25S ribosomal DNA probe (yellow fluorescence) gives prominent hybridisation signals in the distal part of the short arm on the shortest pair of chromosomes in the complement. Strikingly, apart from the rDNA probe, the BAC DNA-based probes do not hybridise to ABR114 chromosomes (Fig. 1e), while the results observed in ABR113 (Fig. 1f) are the perfect amalgam of those obtained separately for *B. distachyon *ABR1 and for ABR114.

Two further clones, 41-E10 (green fluorescence) and 1-H3 (red fluorescence), hybridise to the interstitial regions of two different arms of the second largest and metacentric pair of chromosomes in the complement of *B. distachyon *ABR1 (Fig. 1g). In ABR114, the same clones map subterminally in two different pairs of chromosomes (Fig. 1h), while in ABR113 (Fig. 1i), as in the case of the BAC clones described above (Fig. 1f), the number and chromosomal distribution of BAC-FISH signals makes the sum of the results obtained for *B. distachyon *ABR1 and for ABR114, which further confirms putative allotetraploid status of the accession ABR113.

## Conclusion

The main aim of this study was to develop a method of chromosome preparation that would improve the efficiency of FISH as a technique for comparing closely related species in genera such as *Avena *and *Brachypodium*. Our results show that multi-substrate preparations may offer not only significantly higher throughput but also better reproducibility and reliability of semi-quantitative comparisons of signal intensity than routinely used single-substrate chromosome preparations, especially when hybridised simultaneously to several DNA probes in multicolour FISH experiments. This improvement in efficiency has allowed us to use FISH as an alternative to some molecular analyses, for example in screening repetitive sequences for potential species-specific variation in distribution, and so will accelerate studies aimed at understanding the evolution of complex plant genomes.

## Methods

### Plant material

Seeds of *Avena agadiriana *(2n = 4x = 28; genome AABB), *A. murphyi *(2n = 4x = 28; genome AACC), *A. sterilis *(2n = 6x = 42; genome AACCDD) were from the IGER, UK collection; *Brachypodium distachyon *ABR1 (2n = 2x = 10), *Brachypodium *ssp. ABR114 (2n = 2x = 20) and ABR113 (2n = 4x = 30) were from the seed collection of the University of Wales Aberystwyth, UK.

### Chromosome preparation

Seeds were germinated on moist filter paper in Petri dishes at 20–22°C. Whole seedlings with roots about 2 cm long were immersed in ice-cold water for 24 hours, followed by fixation in 3:1 methanol – glacial acetic acid and stored at -20°C until required. After several 5 minute washes in 0.01 M citric acid – sodium citrate buffer (pH 4.8), excised roots were digested enzymatically at 37°C in a mixture comprising 1% (w/v) cellulase (Calbiochem), 1% (w/v) cellulase ''Onozuka R-10'' (Serva) and 20% (v/v) pectinase (Sigma) for 2 h and 40 min (*Avena*) and 2 h (*Brachypodium*).

Meristems of three different species of *Avena *or *Brachypodium *were dissected from root tips in separate containers containing 45% acetic acid. The number of meristems used depended upon root tip size, with a single meristem being taken for each *Avena *species compared to three for each *Brachypodium *species. Meristems for each individual species were carefully transferred in a small (about 3 μl) volume of 45% acetic acid and arranged on a slide in the form of a triangle (Fig. 1j). A 24 mm × 24 mm coverslip was carefully applied to the slide. After squashing and freezing, coverslips were removed and the preparations were briefly post-fixed in a pre-chilled (-20°C) 3:1 ethanol:glacial acetic acid mix followed by dehydration in absolute ethanol and air drying. The distribution and quality of each of the three substrates on the slide were examined under a phase contrast microscope.

### DNA probes

Probe templates were derived from clones of fragments from three different *Avena *retrotransposon families [13], unpublished), from a 2.3-kb *Cla*I sub-clone of the 25S rDNA coding region of *A. thaliana *[14], or from five different bacterial artificial chromosome (BAC) clones selected from BAC libraries of *B. distachyon *ABR1 and ABR5 15.

Probes were made by single-primer PCR labeling of PCR fragments from plasmid templates (probes KK2.5, KK3.4, KK4.24) or by nick translation of plasmids (BAC clones and 25S rDNA). Briefly, for PCR labelling, the probe template was generated by 18 cycles of PCR. The PCR reaction was then diluted approximately one hundred-fold with fresh reaction mixture containing a single primer and either tetramethyl-rhodamine-5-dUTP (Roche) or digoxigenin-11-dUTP (Roche), and reamplified for a further 11 cycles. Combinatorial probes (yellow fluorescence) were subsequently made by combining probes amplified with both labels. For better visualisation of the 47-F4 clone probe (originally visualised by green fluorescence) pseudo-colouring to purple was used (Fig. 1d and 1f).

### Fluorescence *in situ* hybridisation

FISH was carried out as described previously [12]. Briefly, each PCR-based probe to be hybridised with *Avena *chromosomes was mixed to a final concentration of 2–4 ng/μl in a hybridisation mixture containing: 50% deionised formamide, 2× SSC, 10% (w/v) dextran sulphate, 10 μg/μl sonicated salmon sperm DNA and 0.5% SDS. For BAC-FISH in *Brachypodium*, formamide concentration was reduced to 30%. The probes were denatured separately in hybridisation mixtures (80°C for 10 min), applied to the preparations and denatured combinatorially (70°C for 4.5 min). Hybridisation was carried out for 12–20 h in a humid chamber at 37°C. For *Avena *preparations, post-hybridisation washes were carried out for 10 min either in 10% deionised formamide in 0.1× SSC at 42°C (equivalent to 79% stringency) or, for *Brachypodium*, in 20% deionised formamide in 2× SSC at 37°C (equivalent to 59% stringency). The digoxigenated probes were immunodetected according to standard protocol using FITC-conjugated anti-digoxigenin antibodies (Roche). Finally, preparations were mounted and counterstained in Vectashield (Vector Laboratories) containing 2.5 μg/ml of 4',6-diamidino-2-phenylindole (DAPI). In order to discriminate better the whole chromosome set painted by probe KK3.4 (green fluorescence in Fig. 1a–c), original blue DAPI fluorescence was converted to grey.

### Image acquisition and processing

Images were taken using a Hamamatsu C5810 colour CCD camera attached to an Olympus Provis AX microscope or Hamamatsu ORCA monochromatic CCD camera attached to a Zeiss Axioplan epifluorescence microscope, and colours assigned using Wasabi software (Hamamatsu Photonics). All images were processed uniformly and superimposed using Picture Publisher software (Micrografx/Corel).

## Authors' contributions

R.H. and T.L. designed and executed the laboratory work as well as drafted the manuscript. J.D. participated in planning and execution of the molecular cytogenetic work on *Avena *species. G.J. and M.L. participated in planning the study and contributed in writing the manuscript. All authors read and approved the final manuscript.
